# NHH promotes Sepsis-associated Encephalopathy with the expression of AQP4 in astrocytes through the gut-brain Axis

**DOI:** 10.1186/s12974-024-03135-2

**Published:** 2024-05-27

**Authors:** Lina Zhao, Zhen Zhang, Pei Wang, Nannan Zhang, Hao shen, Hening Wu, Zhiyong Wei, Fei Yang, Yunying Wang, Zhijie Yu, Haibo Li, Zhanfei Hu, Hongyan Zhai, Zhiwei Wang, Fuhong Su, Keliang Xie, Yun Li

**Affiliations:** 1 Department of Critical Care Medicine, Tianjin Medical University General Hospital, Tianjin, 300052 China; 2 Department of Critical Care Medicine, Chifeng Municipal Hospital, Chifeng Clinical Medical College of Inner Mongolia Medical University, Chifeng, 024000 China; 3 Department of Anesthesiology, Chifeng Municipal Hospital, Chifeng Clinical Medical College of Inner Mongolia Medical University, Chifeng, 024000 China; 4 Department of Ultrasound, Tianjin Medical University General Hospital, Tianjin, 300052 China; 5 Experimental Laboratory of the Department of Intensive Care, Erasme Hospital, Université Libre de Bruxelles, Brussels, 1070 Belgium; 6 Department of Anesthesiology, Tianjin Institute of Anesthesiology, Tianjin Medical University General Hospital, Tianjin, 300052 China

**Keywords:** Sepsis-associated encephalopathy, AQP4, GFAP, Gut-brain axis, Astrocyte

## Abstract

Sepsis-associated encephalopathy (SAE) is a significant cause of mortality in patients with sepsis. Despite extensive research, its exact cause remains unclear. Our previous research indicated a relationship between non-hepatic hyperammonemia (NHH) and SAE. This study aimed to investigate the relationship between NHH and SAE and the potential mechanisms causing cognitive impairment. In the in vivo experimental results, there were no significant abnormalities in the livers of mice with moderate cecal ligation and perforation (CLP); however, ammonia levels were elevated in the hippocampal tissue and serum. The ELISA study suggest that fecal microbiota transplantation in CLP mice can reduce ammonia levels. Reduction in ammonia levels improved cognitive dysfunction and neurological impairment in CLP mice through behavioral, neuroimaging, and molecular biology studies. Further studies have shown that ammonia enters the brain to regulate the expression of aquaporins-4 (AQP4) in astrocytes, which may be the mechanism underlying brain dysfunction in CLP mice. The results of the in vitro experiments showed that ammonia up-regulated AQP4 expression in astrocytes, resulting in astrocyte damage. The results of this study suggest that ammonia up-regulates astrocyte AQP4 expression through the gut-brain axis, which may be a potential mechanism for the occurrence of SAE.

**Supplementary Information:**

The online version contains supplementary material available at 10.1186/s12974-024-03135-2.

## Abstract (significance)

This study elucidates the intricate relationship between gut microbiota, ammonia production, and brain dysfunction in sepsis associated encephalopathy.

**Supplementary Information:**

The online version contains supplementary material available at 10.1186/s12974-024-03135-2.

## Introduction

Sepsis-associated encephalopathy (SAE) is a major cause of both short- and long-term mortality in patients with sepsis. Its incidence and mortality rates are increasing globally, posing a serious threat to public health that urgently needs to be solved [1–3]. SAE manifests as cognitive dysfunction and changes in the levels of consciousness and behavior [3]. Although inflammation, mitochondrial dysfunction, and microglial cell activation have been reported as potential mechanisms of SAE, the exact pathogenesis remains unclear, and effective prevention and intervention measures are still lacking.

Ammonia serves as a crucial neurotoxin, playing a significant role in hepatic encephalopathy [4]. Hyperammonemia, stemming from non-hepatic conditions, is increasingly documented among critically ill patients, notably those afflicted by sepsis [5–7]. Our previous investigations have revealed a correlation between non-hepatic hyperammonemia (NHH) and sepsis-associated encephalopathy (SAE). The etiology of NHH in septic patients may stem from factors such as heightened ammonia generation within the intestine and amplified breakdown of amino acids [8, 9]. The gastrointestinal tract, a vital component of the immune system, is susceptible to injury following sepsis, particularly in instances of septic shock. Subsequent to intestinal damage, dysbiosis in the gut microbiota is prone to occur [10]. Ammonia is generated from amino acids in response to intestinal bacteria; Therefore, the disruption of the gut microbiota and the upgrading of amino acid metabolism after sepsis may contribute to NHH production.

Astrocyte edema stands as a pivotal mechanism contributing to the onset of sepsis-associated encephalopathy (SAE) alongside neuronal damage [11]. Additionally, astrocytes play a crucial role in the development of hepatic encephalopathy induced by ammonia [12]. Aquaporin 4 (AQP4) is a specialized membrane-bound channel highly expressed in astrocytes, governing water movement in and out of these cells. Moreover, AQP4 activation elevates astrocyte activity through increased AQP4 expression [11, 13]. Given its significance in astrocytic function, AQP4 emerges as a key player in astrocytic injury.Given its importance in astrocyte function, the study have found AQP4 to be a key player in ammonia-induced astrocyte injury in hepatic encephalopathy [12]. However, it is unclear whether they are involved in the occurrence of SAE.

**Fig. 1:**
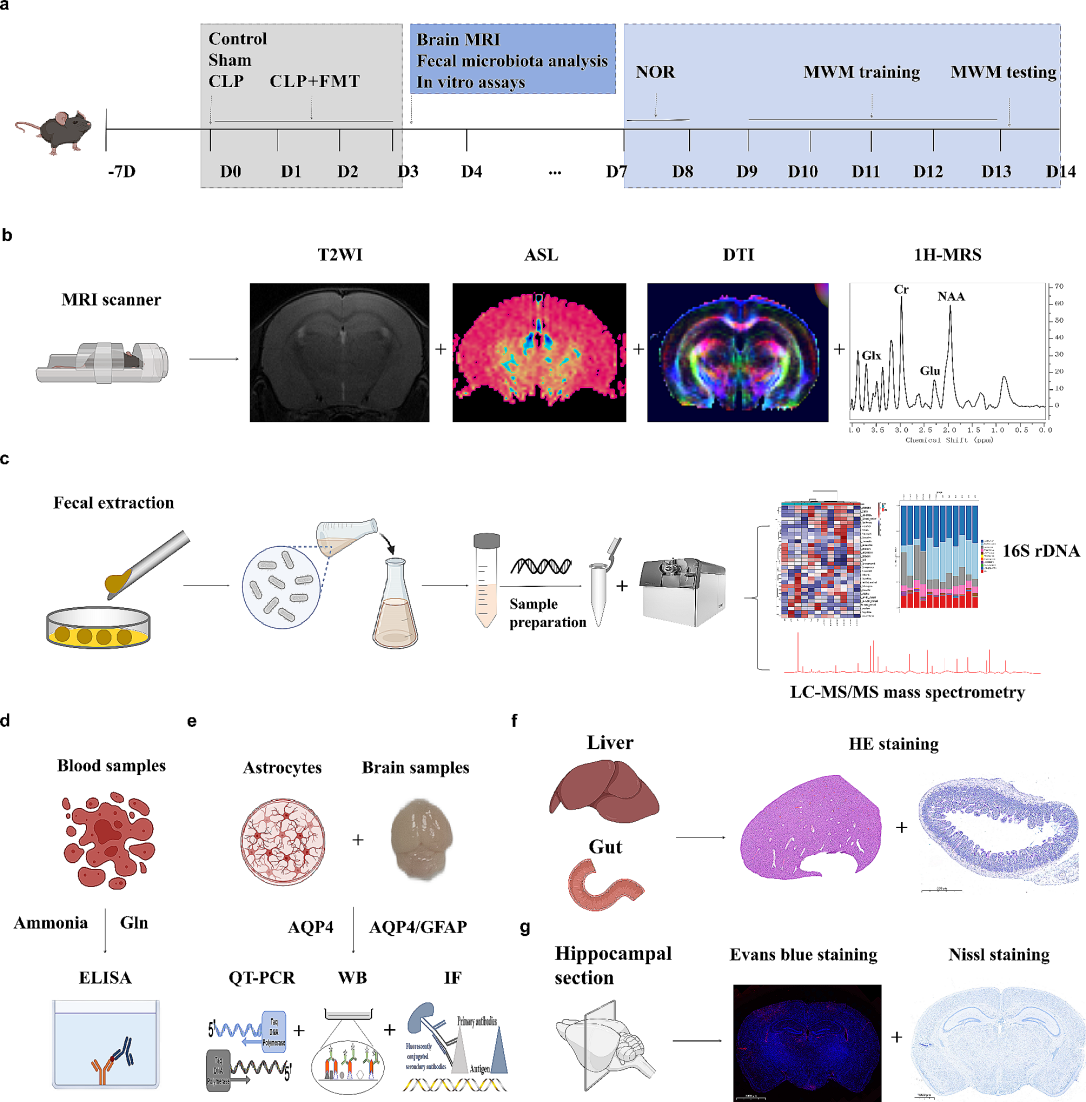
Experimental flow chart. The mice were allowed to acclimatize to the experimental environment for 1 week before the start of the experiment. After CLP or sham surgery in C57BL6/J mice, CLP mice were transplanted with fecal bacteria from the control group mice for 3 consecutive days. (**a**) On the 3 days, the species and abundance of intestinal microbiota were detected (16 S rDNA), neuroimaging (**b**), mettabomics (**c**), including T-2 weighting, DTI, ASL, 1 H-MRS, and molecular biology tests including ELISA (**d**), Western Blot, QT-PCR, immunofluorescence (**e**), HE staining (**f**), Nissl staining and Evans blue staining (**g**). CLP: cecal ligation and perforation; ASL: Arterial spin labeling; DTI: Diffusion Tensor Imaging; 1 H-MRS: proton magnetic resonance spectroscopy; T2WI: T2-weighted imaging; WB: Western Blot. * *p* < 0.05, **p < 0.01, *** *p* < 0.001, *** *p* < 0.0001

This study aimed to explore the origins of ammonia in a mouse model of cecal ligation and puncture (CLP) by examining the potential increase in ammonia-producing bacteria in the intestine, utilizing 16 S rDNA sequencing and metabolomics technology. Pathophysiological alterations and the underlying mechanisms of ammonia-induced brain injury in a CLP mouse model were investigated at both animal and cellular levels. This was achieved through fecal microbiota transplantation and a comprehensive array of analyses including morphological, functional, molecular biology, and neuroimaging techniques (refer to Fig. 1).

## Results

### Elevated levels of NHH in serum and hippocampal tissues of CLP mice

**Fig. 2:**
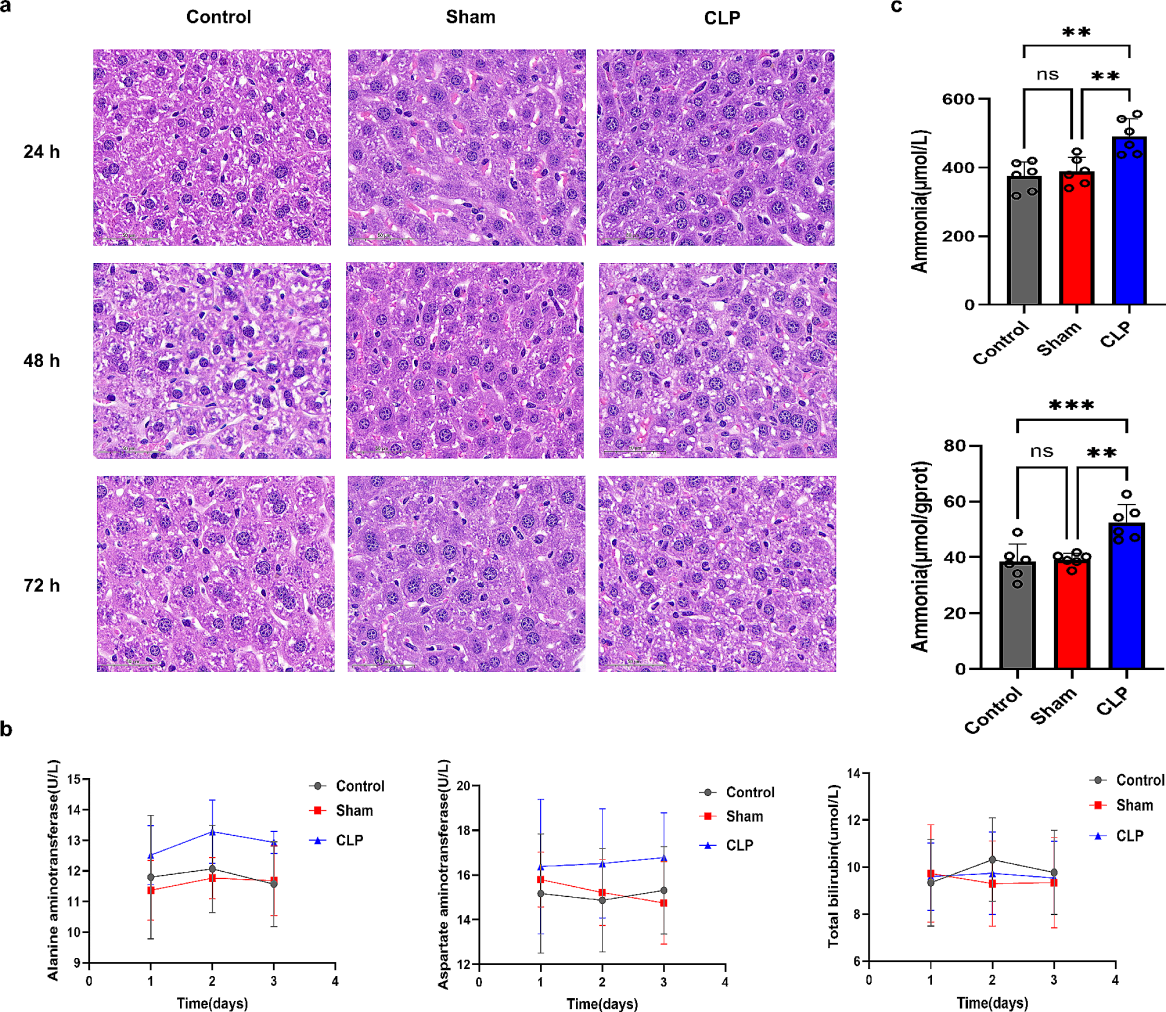
Assessing liver function after mild CLP model in three groups of mice. (**a**) : HE staining of the liver at 24 h, 48 h, 72 h after CLP in the three groups. In the three groups, the hepatocytes of the rats were arranged neatly, and the hepatic lobule structure was intact. (**b**) : CLP caused a slight increase in alanine aminotransferase and aspartate aminotransferase, but the total bilirubin level did not change significantly at 24 h, 48 h, 72 h after CLP. (**c**) : Ammonia levels in serum and hippocampal tissues were significantly higher in the CLP group than in the control and sham groups. ^*^p < 0.05, ^**^p < 0.01, ^***^p < 0.001, ^****^p < 0.0001

Our previous studies found that patients with sepsis and normal liver function had elevated ammonia levels and that the serum ammonia level correlated with impaired consciousness [14]. We speculate that non-hepatic hyperammonemia (NHH) may be a potential mechanism underlying SAE [9]. To verify the change in ammonia in SAE, we used CLP surgery to evaluate the mice’s response to SAE to avoid liver damage, in this study, a moderate CLP model was used [15]. In this study, the mortality rate of moderate CLP model mice was 48%. We also evaluated HE staining and measured alanine aminotransferase (*p* < 0.01), glutamate aminotransferase (*p* < 0.01), and total bilirubin (*p* > 0.05) levels in the livers of the three groups (*n* = 6) at 24 h, 48 h, and 72 h after CLP (Fig. 2a and b). In three groups, CLP resulted in mildly abnormal liver function; however, the hepatocytes of the rats were arranged neatly, the hepatic lobule structure was intact, and there were no significant abnormalities in the liver function indices of the three groups (Fig. 2b). However, serum (*p* < 0.01) and hippocampal (*p* < 0.001) ammonia levels in the CLP group were significantly higher than those in the control and sham groups (Fig. 2c).

### Fecal microbial transplantation in CLP mice can reduce ammonia levels and may be associated with *bacilli*

#### Increased abundance of *bacilli *in CLP mice

**Fig. 3:**
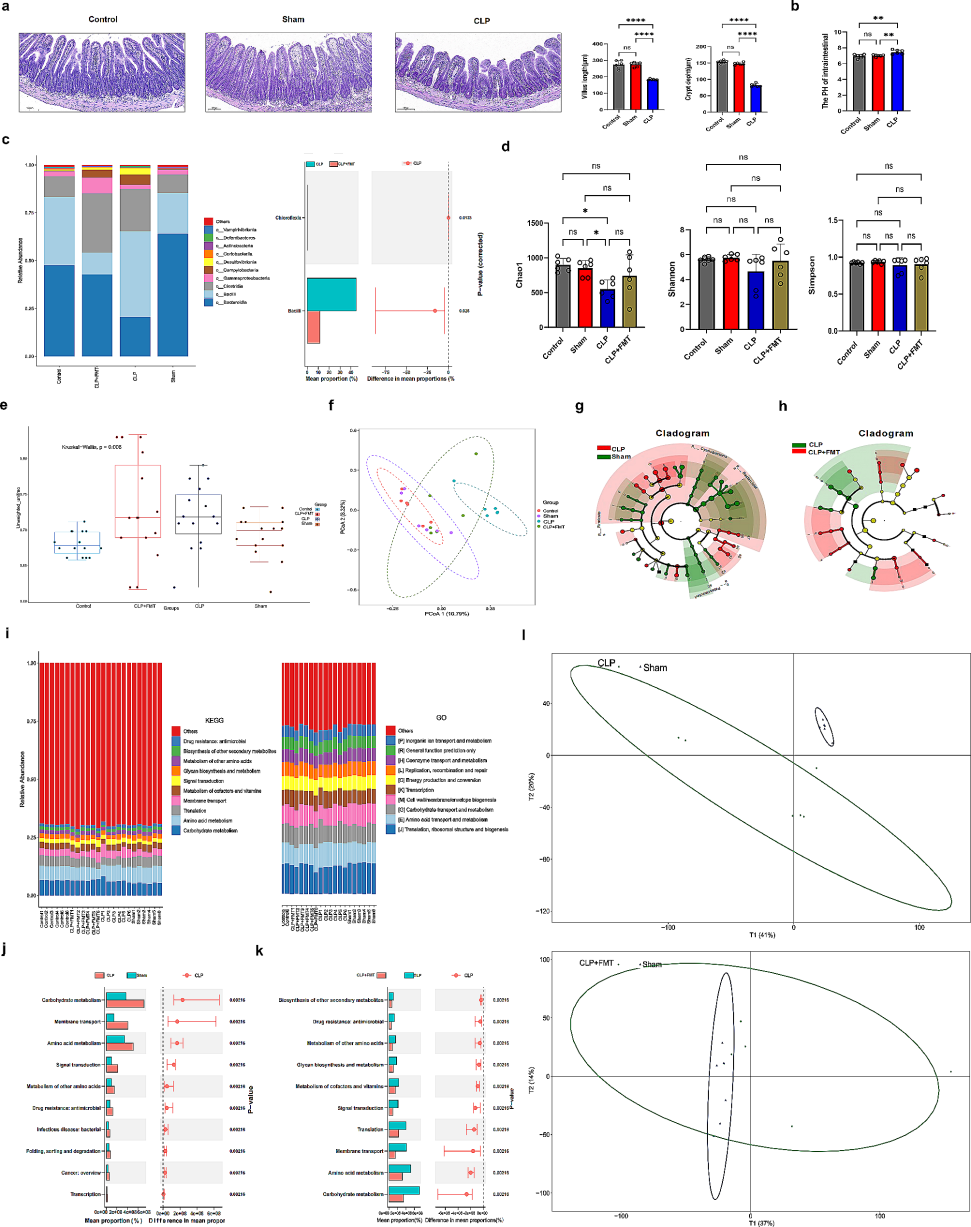
Fecal microbial transplantation in CLP mice can reduce ammonia levels and may be associated with *Bacilli*. (**a**) : HE staining was used to observe the morphological changes of the ileum. (**b**) : Intestinal pH test in the three groups of mice. (**c**) : Observe the changes in microbial species and abundance in each group at the class level through 16 S rDNA technology. (**d**) : Comparison of microbial α diversity among groups. (**e**) and (**f**): Comparison of microbial β diversity among groups. (**g**) : LDA effect size (LEfSe) analysis CLP group compared with sham group. (**h**) : LEfSe analysis CLP group compared with (CLP + FMT) group. (**i**) : The functional annotation and differential analyses by KEGG and Go. (**j**) and (**k**) : Functional difference was analyzed for each group. Figure 3l: PCA analysis of microbiota function. KEGG: The Kyoto Encyclopedia of Genes and Genomes; GO: the Gene Ontology. ^*^p < 0.05, ^**^p < 0.01, ^***^p < 0.001, ^****^p < 0.0001

After the CLP model was established, the fecal microorganisms of the mice in the control group were transplanted into the CLP mice for 3 consecutive days. HE staining was used to observe the morphological changes in the ileum and intestinal pH test in the three groups of mice (*n* = 6). After CLP, the ileum villus length and crypt depth were decreased, and pH was increased compared to the control and sham groups (*p* < 0.0001 or *p* < 0.01) (Fig. 3a and b). CLP disrupts the intestinal structure of mice and pH. After fecal microbe transplantation (FMT), the feces of mice in each group (*n* = 6) were collected, and 16 S rDNA sequencing technology was used to observe the changes in microbial species and abundance in each group. After CLP, at the class level, the most abundant microbes of *Bacteroidia* decreased, while *Bacilli* increased compared to the control group and sham group. After FMT, the abundance of Bacteroidia microbes increased, and *Bacilli* decreased in the community plot (*p* < 0.05) (Fig. 3c). The diversity of the microbiota was significantly lower in the CLP group than in the control and sham groups, as determined by the Chao1 index (*p* < 0.05); however, there were no significant differences in the Shannon and Simpson indices (Fig. 3d). Additionally, the analysis of microbiome beta diversity using Unweighted Unifrac and principal coordinate analysis (PCoA) indicated significant differences in community components between the CLP group and the other three groups, indicating that CLP affected gut microbiota composition (*p* < 0.01) (Fig. 3e and f). The LDA effect size (LEfSe) analysis showed that the key bacteria in the sham group were *Bacteroidales, Muribaculaceae, Erysipelotrichaceae, Allobaculum, Prevotellaceae, and Alloprevotella*. The key bacteria in CLP mice were *Bacilli, Clostridium, Firmicutes, Lactobacillales, Enterobacteriales, Streptococcus*, and *Enterococcus* were predominant (Fig. 3g and Supplementary Materials 1). Fecal transplantation resulted in decreased relative abundance of *Bacilli* and *Clostridium* and increased relative abundance of *Rhodospirillales*, *Moraxellaceae, Clostridia_vadinBB60_group*, *Gastranaerophilales*, and *Clostridia_UCG_014* in CLP group (Fig. 3h and Supplementary Materials 2). These results showed that CLP significantly altered the type and abundance of the intestinal flora.

Functional annotation and differential analyses were conducted to investigate the effects of modified gut microbiota. The Kyoto Encyclopedia of Genes and Genomes (KEGG) and Gene Ontology (GO) functional annotation results showed that the relative abundance values of amino acid metabolism in the CLP group were higher than those in the other groups(Fig. 3i). Functional difference analysis also showed that “Carbohydrate metabolism, “Amino acid metabolism,” “ Metabolism of other amino acids”, and “Infectious disease: bacterial” were stimulated in the CLP group (Fig. 3j) (*p* < 0.05) after FMT, they were suppressed (Fig. 3k) (*p* < 0.05). Principal Component Analysis (PCA) analysis of microbiota function showed that there was a significant difference in microbiota function between the CLP and sham groups, and microbiota function was similar after FMT (Fig. 3l). Therefore, it is speculated that amino acid levels may be related to changes in the intestinal flora.

### FMT reduced amino acid metabolism and ammonia levels in CLP mice

**Fig. 4:**
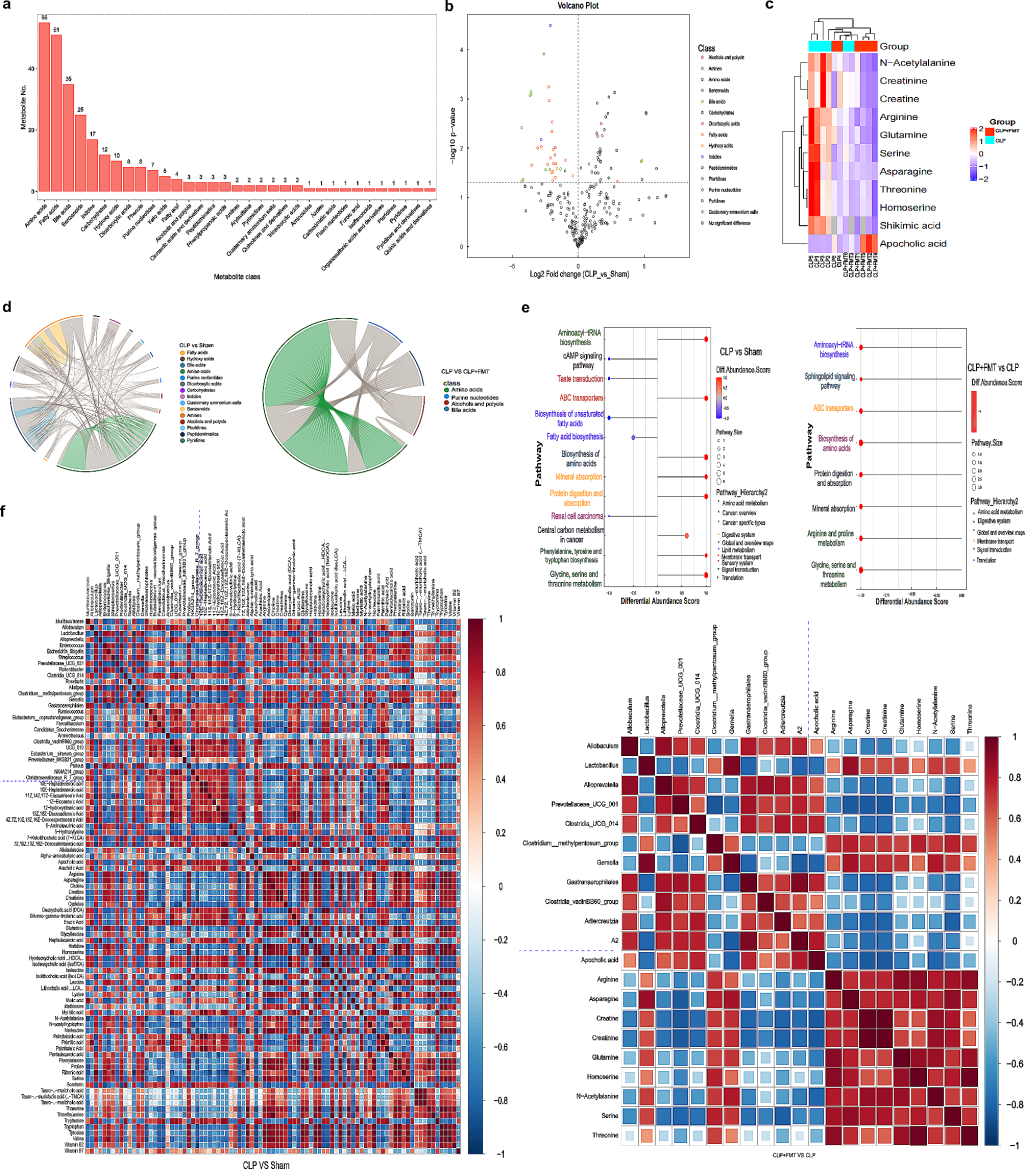
Fecal microbial transplantation reduced amino acid metabolism and ammonia levels in CLP mice. *Bacilli* has a significant correlation with amino acid metabolism and ammonia. (**a**) : Metabolomic analysis by LC-MS/MS showed that amino acid counts accounted for 55% of all identified metabolite numbers. (**b**) : The amino acid levels in the CLP group were significantly higher than those in the sham group in Volcano plots. (**c**) : after fecal microbial transplantation, the amino acid substance of the CLP group was significantly reduced in volcano plots and heat maps. (**d**) : The metabolite comparison of the chord diagrams of the four groups. (**e**) : KEGG pathway analysis of metabolites in CLP group, sham group, CLP + FMT group. (**f**) : The association between intestinal microbes and metabolic products in the CLP group, sham group, CLP + FMT group. ^*^p < 0.05, ^**^p < 0.01, ^***^p < 0.001, ^****^p < 0.0001

**Fig. 5:**
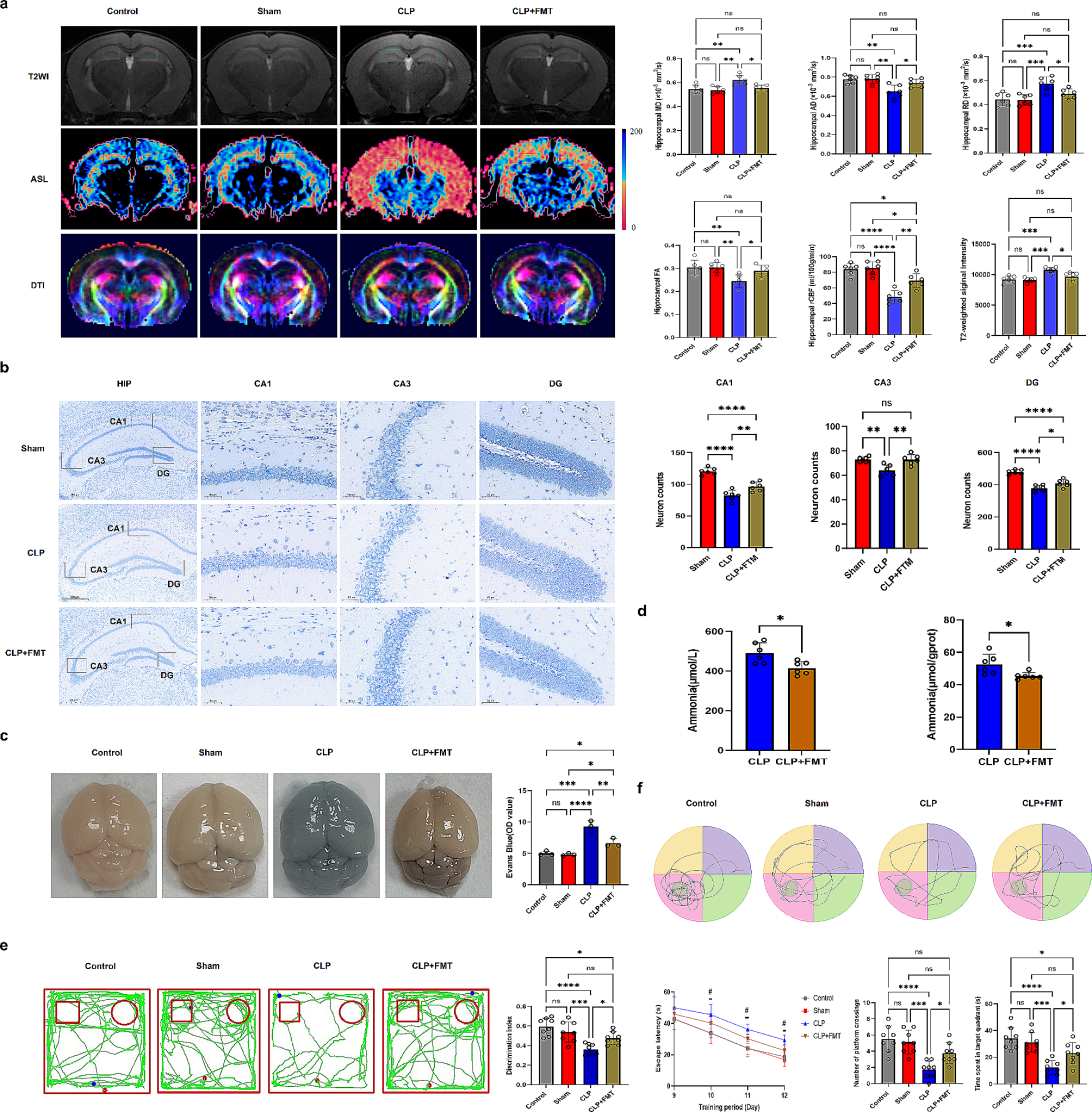
The decrease in ammonia levels improved cognitive dysfunction and neuronal damage in CLP mice. (**a**) : T2WI, ASL, and DTI were perfected for 72 h after CLP surgery in each group. The red box shows the comparison of T2WI signal density and hippocampal rCBF. (**b**) : The Nissl staining of hippocampal tissues(DG, CA1, CA3) was perfected in each group. (**c**) : Evans blue dye extravasation was used to assess changes in vascular permeability in each group. (**d**) : Ammonia levels in the hippocampus and serum were compared between the CLP group and the CLP + FMT group. (**e**) : The novel object recognition was completed in each group of mice. (**f**) : The Morris Water Maze was completed in each group of mice.T2WI: T2-weighted imaging ^*^p < 0.05, ^**^p < 0.01, ^***^p < 0.001, ^****^p < 0.0001

Metabolomic analysis by LC-MS/MS showed that amino acids accounted for 55% of all identified metabolites (Fig. 4a). The amino acid levels in the CLP group were significantly higher than those in the sham group, with Log2FC values ranging from 1.15 to 60.87 in volcano plots (*p* < 0.05). The identified amino acids included 5-Aminolevulinic acid, 5-Hydroxylysine, Alloisoleucine, Alpha-aminobutyric acid, Arginine, Asparagine, Glutamine, Histidine, Homoserine, Lysine, Methionine, N-Acetylalanine, Phenylalanine, Proline, Serine, Threonine, Tryptophan, Tyrosine, and Valine (Fig. 4b). After FMT, the amino acid substance of the CLP group was significantly reduced, with Log2FC values ranging from 0.10 to 0.49 (*p* < 0.05), the reduced of amino acids included Arginine, Asparagine, Creatine, Glutamine, Homoserine, N-acetylalanine, Serine, and Threonine (Fig. 4c). Additionally, the chord diagrams the four groups further confirmed that there was a significant difference in amino acid levels (*p* < 0.05), with a correlation between amino acids (*r* > 0.8) (Fig. 4d, Supplementary material 3). KEGG pathway analysis of metabolites showed that amino acid metabolism increased after CLP, and the difference in abundance was higher than that of the sham group, including phenylalanine, tyrosine, and tryptophan biosynthesis; glycine, serine, and threonine metabolism after Fecal microbial transplantation, arginine, and proline metabolism; and glycine, serine, and threonine metabolism were reduced (Fig. 4e). Additionally, after FMT in CLP mice, ammonia levels in the serum and hippocampal tissues decreased (Fig. 5d). These results showed that FMT reduced amino acid metabolism and ammonia levels in the CLP group. Amino acid metabolism is the primary source of ammonia.

#### *Bacilli *has a significant correlation with amino acid metabolism and ammonia

In this study, the association between intestinal microbes and metabolic products was analyzed in each group. The *Bacilli* of CLP group and Sham group differentiating bacteria is positively correlated with the metabolism of Arginine, Asparagine, Cysteine, Glutamine, Glycylleucine, Histidine, Homoserine, Isoleucine, Lysine, N − Acetylalanine, N − acetyltryptophan, Norleucine, Phenylalanine, Proline, Serine, Tyrosine, and Threonine. The *Bacilli* of CLP and Sham group differentiating bacteria is positively correlated with the metabolism of Arginine, Asparagine, Creatine, Creatinine, Glutamine, Homoserine, N − Acetylalanine, Serine, Threonine (Fig. 4f). Therefore, we speculate that the increase in *Bacilli* after CLP may be related to the occurrence of NHH.

### Decreased ammonia levels improved cognitive dysfunction and neuronal damage in CLP mice

#### Neuroimaging

T2-weighted Arterial spin labeling (ASL), Diffusion Tensor Imaging (DTI), and Proton magnetic resonance spectroscopy (1 H-MRS) were performed 72 h after CLP surgery (*n* = 6). T2-weighted test results showed that the signal intensity of the CLP group was significantly higher than that of the sham and control groups, which showed an increase in water content in mouse brain tissue after CLP. Furthermore, FMT reduced ammonia levels, resulting in decreased water content of brain tissue in CLP mice (*p* < 0.001 or *p* < 0.05). ASL examination results showed a significant decrease in blood flow in the hippocampal region of CLP mice. After FMT in CLP mice, cerebral blood flow improved (*p* < 0.01 or *p* < 0.05 or *p* < 0.0001). DTI test results showed that the fractional anisotropy (FA) and Axial Diffusion (AD) of hippocampal tissues in CLP mice were significantly higher than those in the sham and control groups, while the Radical Diffusion (RD) and Mean Diffusivity (MD) of hippocampal tissues in CLP mice were significantly lower than those in the sham and control groups (*p* < 0.01, *p* < 0.05, or *p* < 0.0001), suggesting that CLP could lead to impaired nerve fiber integrity in mice, with improved nerve fiber integrity observed after reducing ammonia level (Fig. 5a). Imaging studies have shown that CLP can lead to cerebral edema, decreased cerebral blood flow, and destruction of the nerve fiber bundle integrity. However, after FMT and reduction of ammonia levels in mice, cerebral edema, decreased cerebral blood flow, and nerve fiber incompleteness improved.

### Nissl staining of hippocampal tissues

Nissl staining of hippocampal tissues was performed 72 h after CLP surgery (*n* = 6). The results showed that CLP can lead to a decrease in hippocampal neuronal cells. Moreover, after reducing ammonia levels, the damage caused by CLP to neuronal cells was mitigated (*p* < 0.01, *p* < 0.05, and *p* < 0.0001, respectively) (Fig. 5b).

### Blood-brain barrier permeability

Evans blue dye extravasation was used to evaluate alterations in vascular permeability 72 h after CLP (*n* = 6). Evans blue extravasation into the entire brain was measured in the CLP group. The large increase in Evans blue extravasation in the CLP mice was significantly reduced in the CLP + FMT mice (Fig. 5c). Pathological studies have shown that CLP not only damages neuronal cells but also destroys the blood-brain barrier and reduces brain damage caused by CLP after reducing ammonia levels (*p* < 0.01, *p* < 0.05, and *p* < 0.0001).

### Behavioral

On days 7 and 8 after CLP [35], new-object recognition was evaluated for cognitive memory in each mouse group (*n* = 8). The results showed a reduction in the discrimination index of mice after CLP, after FMT in the CLP group, the discrimination index improved (*p* < 0.001, *p* < 0.05, and *p* < 0.0001, respectively) (Fig. 5e). From days 9 to 13 after CLP, the Morris Water Maze was employed to evaluate the spatial learning and memory function of each group of mice. The number of platform crossings and the time spent in the target quadrant in the CLP group were significantly reduced compared to the sham group and the control group, while the escape latency was prolonged (*p* < 0.001, *p* < 0.05, and *p* < 0.0001, respectively) (Fig. 5f). The behavioral study results showed impaired cognitive function of mice after CLP, and the reduction of ammonia levels could improve the cognitive function of CLP mice, thereby further confirming the success of the SAE model.

### Ammonia up-regulates astrocytes AQP4 may be the mechanism of SAE

**Fig. 6:**
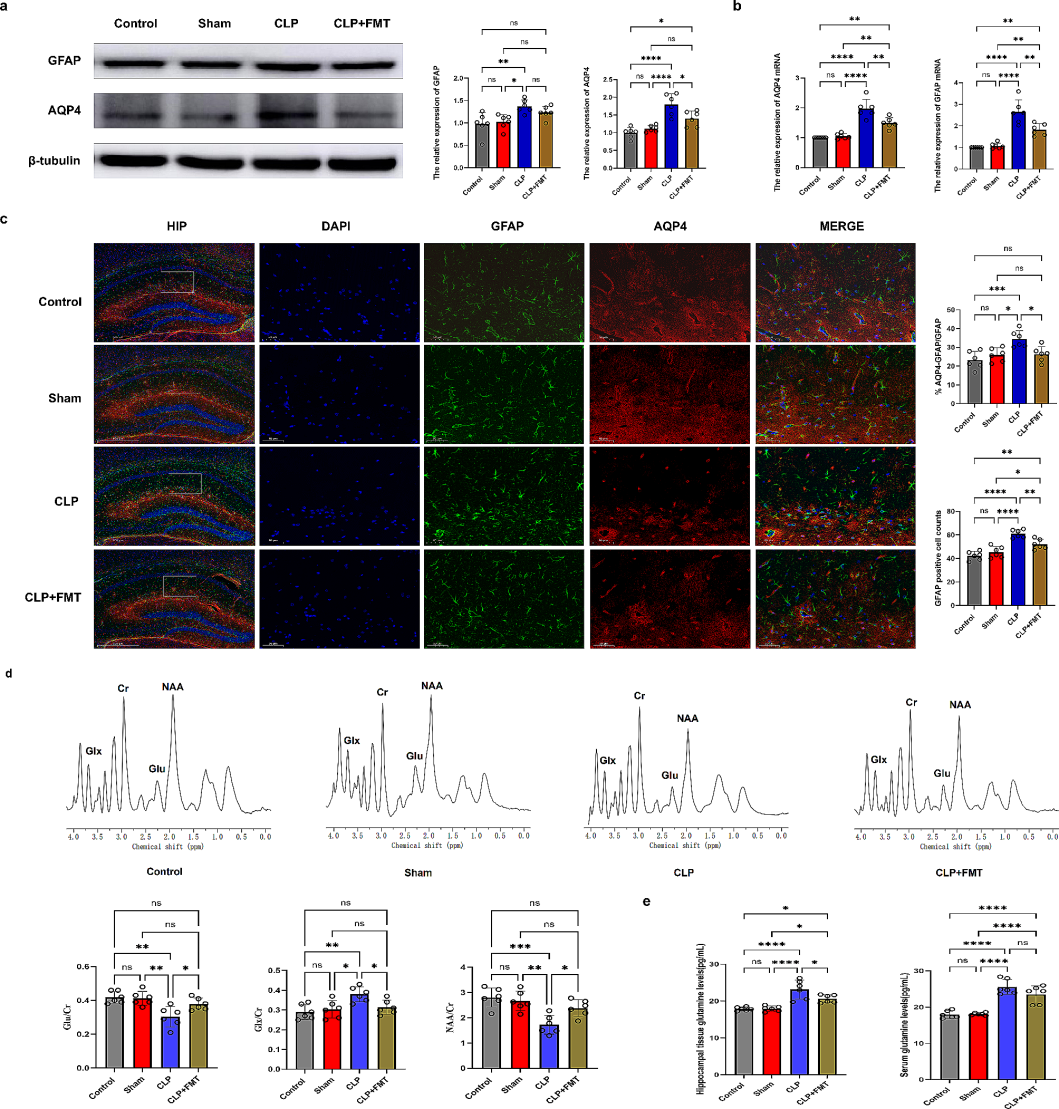
Ammonia up-regulates astrocytes AQP4 may be the mechanism of SAE. (**a**) : The expression of AQP4 and GFAP protein in hippocampal tissue was detected by western blot. (**b**) : The expression of AQP4 and GFAP gene in hippocampal tissue was detected by QT-PCR. (**c**) : Hippocampal tissue AQP4 co-expression with GFAP was detected by immunofluorescence. (**d**) : The changes in the level of metabolites in the hippocampus of mice in each group were detected by 1 H-MRS. (**e**) : Comparison of glutamine levels in serum and hippocampal tissues of mice in each group. ^*^p < 0.05, ^**^p < 0.01, ^***^p < 0.001, ^****^p < 0.0001

The western blot (WB) analysis (*n* = 6) and Quantitative Real-time PCR (QT-PCR) (*n* = 6), immunofluorescence staining (*n* = 6) results showed that 72 h after CLP, the expression of AQP4 and Glial fibrillary acidic protein (GFAP) proteins and genes, as well as the number of GFAP-positive cells, and the positive rate of AQP4 cell number as a proportion of GFAP cell number in hippocampal tissues were significantly increased (*p* < 0.0001 or *p* < 0.001 or *p* < 0.01 or *p* < 0.05) (Fig. 6a, b and c). Additionally, 1 H- MRS study found that after CLP, the levels of glutamate(Glu)/creatine(Cr) and N-acetyl aspartate(NAA)/Cr in the hippocampus of mice were decreased, indicating neuronal damage, while the level of Glx (glutamate + glutamine)/Cr was increased. After FMT, the serum ammonia level was decreased, neuronal damage was improved, and the level of Glx/Cr was decreased (*p* < 0.0001 or *p* < 0.001 or *p* < 0.01 or *p* < 0.05) (Fig. 6d). Simultaneously, the use of the Elisa method to detect glutamine levels in serum and hippocampal tissues of mice in each group was consistent with the results of 1 H-MRS (Fig. 6e). A previous study had found that ammonia enters the brain and binds to GLU to produce glutamine, further activating astrocytic AQP4 expression and leading to brain damage [16]. To further verify the mechanism of action of ammonia in the brains of CLP-treated mice, cellular experiments were performed.

### Ammonia up-regulates astrocytes’ AQP4 expression by activating astrocytes, resulting in astrocytes damage

**Fig. 7:**
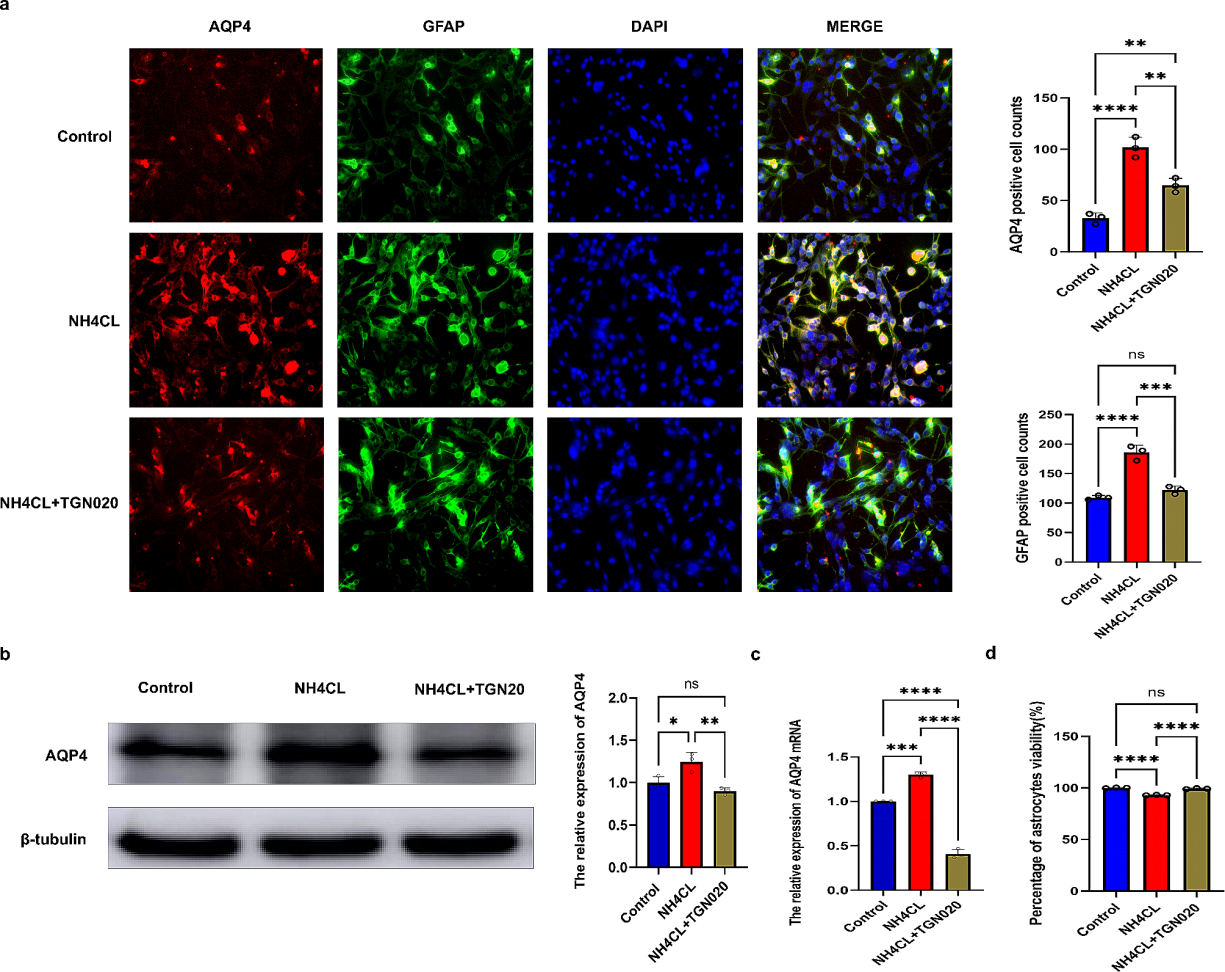
Ammonia up-regulates astrocytes’ AQP4 expression by activating astrocytes, resulting in astrocytes damage. (**a**) : Immunofluorescence co-expression of AQP4 and GFAP in astrocytes in each group. (**b**): AQP4 protein expression in astrocytes was detected by western blot. (**c**) : AQP4 gene expression in astrocytes was detected by QT-PCR. (**d**): Astrocyte viability was measured. ^*^p < 0.05, ^**^p < 0.01, ^***^p < 0.001, ^****^p < 0.0001

Astrocytes were treated with NH_4_Cl for 72 h (*n* = 3) to test the effect of ammonia on them. Immunofluorescence results showed that astrocytes were activated, and the number of GFAP-positive cells increased significantly compared to the control group (Fig. 7a). Subsequently, the number of AQP4 and GFAP co-expressed cells also increased (Fig. 7a) (*p* < 0.0001 or *p* < 0.001 or *p* < 0.01). Additionally, compared with the control group, astrocyte viability decreased after NH_4_Cl treatment (Fig. 7d). WB and QT-PCR results showed that the expression levels of AQP4 protein and gene increased after NH4Cl stimulation (*p* < 0.0001, *p* < 0.001, *p* < 0.01, and *p* < 0.05, respectively) (Fig. 7b and c). To further demonstrate the effect of AQP4 on astrocytes, we treated astrocytes with the AQP4 inhibitor TGN20, which showed decreased astrocyte activation levels, decreased GFAP-positive cell numbers, decreased levels of co-expression with AQP4, and increased astrocyte viability (Fig. 7a and d). Therefore, cell experiments confirmed that ammonia can induce an increase in AQP4 expression and activate astrocytes.

## Discussion

The incidence of NHH is as high as 76% in critically ill patients [17]. Particularly, it has a high incidence among patients with sepsis in critical care settings [6, 8, 18]. In this study, NHH also occurred in the acute phase of CLP in mice, and the results were consistent with those observed in patients with sepsis. Ammonia is primarily produced by the urease generated by intestinal bacteria, serving as the main source of serum ammonia.

After sepsis, the intestinal barrier becomes susceptible to damage, and such damage can contribute to the progression of sepsis [19, 20]. Consistent with these findings, in this study, the ileum villus length and crypt depth were decreased, and pH was increased after CLP, indicating disruption of the intestinal structure. Moreover, CLP led to intestinal dysbiosis, as evidenced by significant changes in the species and abundance of intestinal microbiota. Specifically, the Chao1 index and beta diversity in the CLP group were significantly different from those in the Sham group and the control group. The main manifestations included an increase in *Bacteroidia* and a decrease in *Bacilli*. The gut microbiota is closely related to changes in ammonia levels [21]. In this study, both the serum ammonia and brain tissue ammonia levels of the CLP group decreased after Fecal microbial transplantation, aimed at restoring the normal intestinal microbial state of the CLP group mice. Metabolomic analysis by liquid chromatography-tandem mass spectrometry showed that the amino acid levels in the CLP group were significantly higher than those in the sham group. Moreover, the level of amino acid metabolism in the CLP group decreased after fecal transplantation. Enrichment and correlation analyses showed that *Bacilli* regulate amino acid metabolism and are closely related. *Bacilli*, known as a representative urease-producing strain [22], promotes an increase in ammonia levels. Therefore, we hypothesize that the increase in NHH levels in CLP model mice is associated with the most significant change in the abundance of *Bacilli*. Based on the changes in amino acid levels observed in CLP mice model and after Fecal microbial transplantation, as well as the correlation analysis results between gut microbiota and metabolomics, we deduced that *Bacilli* regulate the increased metabolism of Arginine, Asparagine, Glutamine, Homoserine, N-Acetylalanine, Serine, and Threonine. These pathways represent potential mechanisms underlying the occurrence of NHH in CLP mice model.

Ammonia is an important neurotoxic substance, which is also an important mechanism for the occurrence of hepatic encephalopathy [4]. In recent years, the rise in ammonia levels unrelated to liver dysfunction has received increased attention due to its effect on the nervous system. Ammonia produced by the gut microbiome can help buffer stress in the host, thereby providing a gut-brain signaling basis for emotional behavior [21]. In this study, we observed increased ammonia levels in CLP model mice despite their normal liver function. However, after Fecal microbial transplantation, ammonia levels were reduced in CLP model mice. This reduction was associated with improved cognitive function (MWM and NOR), cerebral edema (T2-weighted imaging, T2WI), cerebral ischemia (ASL), improvement of nerve fiber structural integrity (DTI), blood-brain barrier permeability (Evans blue), and increased neuronal cells in the hippocampus (Nissl staining). Therefore, NHH also plays an important role in brain injury in CLP mice based on multimodal brain evaluation in CLP mouse models. Several studies have shown that the gut-brain axis plays an important role in the pathogenesis of SAE, demonstrating that the gut microbiota facilitates SAE susceptibility [23, 24]. However, in the present study, we found that gut microbes increased ammonia levels by regulating amino acid metabolism, resulting in nervous system damage in SAE mice.

**Fig. 8:**
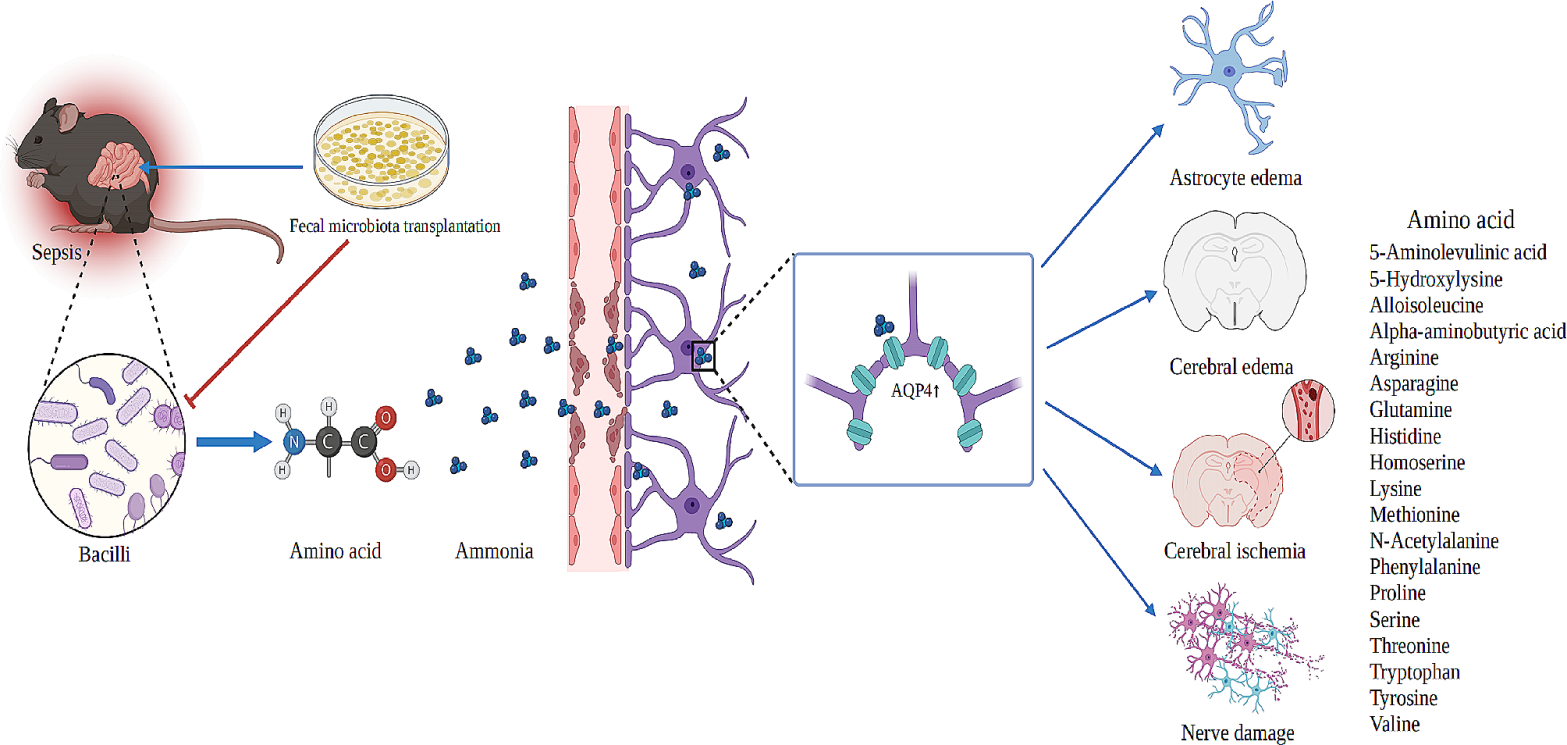
A diagram of the mechanism of NHH leads to SAE. After sepsis, ammonia-producing *Bacilli* increase in the intestine, promoting the breakdown of amino acids, ammonia increases, ammonia enters the brain and aggregates in astrocytes, up-regulating the expression of astrocyte hydration protein AQP4, resulting in astrocyte activation, edema, promoting cerebral edema, cerebral ischemia, and nerve damage, leads to the occurrence of SAE

After ammonia enters the brain tissue, it is rapidly taken up by astrocytes and synthesized into glutamine by glutamine synthetase. The inhibition of astrocyte proliferation by ammonia is mediated by l-methionine sulfoximine, oxidative stress, and p38(MAPK) -MAPK-dependent activation of p53 in hepatic encephalopathy [25]. Additionally, the research has shown that ammonia causes neuronal disinhibition and seizures by disrupting astrocyte potassium buffering [26]. However, the mechanism of ammonia in SAE is still unclear. In this study, cerebral edema occurred in CLP mice in T2-weighted imaging images of CLP mouse models, and cerebral edema was reduced after reducing ammonia level expression. Astrocytic edema is an important mechanism of cerebral edema. AQP4 in astrocytes is the main protein controlling the inflow and outflow of water [27]. In this study, the expression of GFAP and AQP4 proteins and genes in CLP mouse models increased, indicating that astrocytes and aquaporin cells were activated in CLP mice. Additionally, the AQP4-GFAP/GFAP ratio in CLP mice was significantly increased, and GFAP and AQP4 were co-expressed. 1 H- MRI studies showed that after CLP, the levels of Glu/Cr and NAA/C in the brain of mice were decreased, indicating neuronal damage. Meanwhile, the levels of glutamine were increased. However, after reducing ammonia levels, the expression of GFAP and AQP4 proteins, genes, and cell counts of CLP mice decreased, and neuronal damage improved. Additionally, although there was no significant difference in glutamine levels, it was reduced in the serum and hippocampus. Based on these findings, we speculate that NHH induces the upregulation of AQP4 expression in astrocytes, leading to astrocyte swelling, reduced cerebral blood flow, and neuronal damage, potentially contributing to the mechanism of brain injury in CLP mice (Fig. 8).

To further clarify the mechanism of NHH-induced brain injury in the mouse model of CLP, cell experiments were performed. The results showed that the number of GFAP and AQP4 co-expressing cells increased significantly after the NH4Cl culture of astrocytes. Furthermore, GFAP expression was reduced when the AQP4 inhibitor TGN020 was added. These findings suggest that ammonia entry into the brain leads to the upregulation of AQP4 expression in astrocytes, which represents an important mechanism underlying brain injury. Ammonia is reportedly involved in the pathogenesis of hepatic encephalopathy by regulating astrocytic AQP4 expression in the brain [28]. Zhu DD and colleagues found that AQP4 aggravates cognitive impairment in SEA by inhibiting Nav1.6-mediated astrocyte autophagy [11]. In this study, we found that ammonia entering the brain increased the expression of AQP4 in astrocytes in a mouse model of CLP. However, the potential mechanism by which AQP4 causes brain injury in this model remains unclear and requires further exploration in future research.

Limitations of our study including (1) healthy rodent animals do not represent the patients; (2) the CLP model was used in the SAE model of mice, which showed abdominal infection, and the infection sites of SAE patients in clinical practice often included the brain, lungs, abdomen, bloodstream infection and other sites, therefore, the results of this study may be more relevant to those of patients with SAE with abdominal infection in the clinic. (3) clinical relevance including the fluid resucistaiton, antibiotics, vasopressor and source control in this model were not reflected, therefore, the results of this study may be biased against patients with SAE in clinical practice. In this study, we wanted to observe the effect of CLP on intestinal microbial species, but, antibiotics would a significant effect intestinal microbial species [33], therefore in this study did not use antibiotic refer to other literature related to SAE [11, 34]. (4) the changes in ammonia levels and imaging in CLP were not dynamically monitored and observed in the long term.

In summary, the disturbance of the intestinal flora after sepsis, characterized by an increase in urease-producing *bacilli*, leads to increased amino acid metabolism and ammonia production. These changes result in the upregulation of AQP4 levels in astrocytes, facilitating the entry of ammonia into the brain. Consequently, CLP mice exhibit cerebral edema, impaired structural integrity of nerve fibers, neuronal cell damage, and cognitive dysfunction, potentially representing an underlying mechanism for the occurrence of SAE. This study provides new insights into the pathological mechanisms and potential new drug targets for the treatment of SAE.

## Materials and methods

### Animal

The experimental procedures and animal care protocols were approved by the Committee on Ethical Use of Animals of Tianjin Medical University General Hospital. The experimental procedures followed the National Institutes of Health Guidelines for the Care and Use of Laboratory Animals. Male C57BL/6J mice, aged 8–10 weeks and weighing 20–25 g, were obtained from the Specific Pathogen-Free (SPF) Model Animal Center of the Military Medical Science Academy in Beijing, China. The mice were categorized into control, sham, CLP, and CLP + FMT groups.

### CLP model

The experiment utilized the classical CLP model [29]. Briefly, after administering 2% isoflurane anesthesia and disinfecting the skin, a 1-cm incision was made along the abdominal midline to reveal the cecum. The cecum ligation was about 50% ligated between the distal pole and base, 21G needle size was used to passed through the cecum, to induce moderate sepsis. The incision was closed using a sterile 6 − 0 silk suture. Mice subjected to the sham operation had their cecum exposed in the same manner as those subjected to CLP, but the cecum was neither ligated nor punctured. The mice in the control group did not undergo surgery. On the day of surgery, fresh FMT was prepared by collecting the cecal contents from healthy mice. On postoperative days one, two, and three, the selected CLP group of mice was administered 2 mL of FMT via rectal enema using a blunt vein irrigation cannula. The FMT protocol was based on the study of Kim et al. [20].

### Genomic DNA extraction

Genomic DNA was extracted from the samples using an OMEGA Soil DNA Kit (D5625-01) (Omega Bio-Tek, Norcross, GA, USA). The selected V3-V4 variable region was amplified by PCR using specific primers with barcodes and high-fidelity DNA polymerase. The PCR products were detected by 2% agarose gel electrophoresis, and the fragments of interest were recollected using the Quant-IT Pico Green dsDNA Assay Kit. The PCR-amplified recovered products were assayed and quantified using a microplate reader (BioTek, FLx800) and a fluorescence quantitative system. Library-building reagents were obtained using Illumina TruSeq Nano DNA LT Library Prep Kit Box. Quality assessment of the built library was performed using a Bioanalyzer 2100 and Promega Quant Fluor.

### 16 S rDNA processing and analysis

The 16 S analysis uses the Silva (version138) database for species comparison and annotation. Qiime2 software was used to calculate the α β diversity indices. The analysis included the Wilcoxon rank sum test for assessing differences in α diversity (Shannon, Simpson, Chao 1) and β diversity (based on Weighted Unifrac and Unweighted U distances). Differences in community composition were analyzed using principal coordinate analysis (PCoA) or PCA.

LEfSe (LDA Effect Siz) analysis was conducted to identify species significantly impacting the differentiation of samples between groups. STAMP difference analysis was used to compare the abundance of species and the differential species functions between two sets of samples (Wilcox test analysis) or multiple sets of samples (Kruskal–Wallis test analysis). Piecrust software was used to analyze functional differences between different groups by comparing species composition information obtained from 16 S sequencing data, including KEGG and COG.

### LC/MS untargeted metabolomics analysis

Following sample pretreatment, metabolite analysis was conducted using a UHPLC system (1290 Infinity LC, Agilent Technologies) combined with a QTRAP MS instrument (6500, Scitex) at Shanghai Applied Protein Technology Co., Ltd. The analytes were separated using HILIC and C18 columns in UPLC with specific column specifications (HILIC: Waters UPLC BEH Amide column, 2.1 mm × 100 mm, 1.7 μm; C18: Waters UPLC BEH C18-2.1 × 100 mm, 1.7 μm). Multiquanta was used for quantitative data processing. The processed data were uploaded to SIMCA-P (version 14.1, Umetrics, Umea, Sweden) for multivariate data analysis, which included Pareto-scaled principal component analysis (PCA) and other analyses.

### Magnetic resonance imaging (MRI)

MRI of each group of mice was conducted using a 9.4T magnetic resonance small animal scanner (Bruker Bio spin, Germany) at Tianjin Medical University General Hospital. In this experiment, MRI techniques, including T2-weighted imaging, ASL, TDI, and 1 H-MRS, were used to observe changes in cerebral edema, cerebral blood flow, nerve fiber integrity, and metabolites in each group of mice.

### T2-weighted imaging

The Parameter settings for T2-weighted imaging (T2WI) were as follows: repetition time (TR)/echo time (TE) = 4808/33 ms, field of view (FOV) = 20 × 20 mm^2^, slice thickness = 0.5 mm, number of averages (NA) = 2, and slices = 50.

### 1 H-MRS

The parameter settings for 1 H-MRS were as follows: RT/TE = 1500/16.5 ms, averages = 256, target voxel of interest, 4 × 1 × 2 mm^3^. Data analyses were performed using the Bruker Para Vision 360 and Mestre Nova software (V12.0.0, Mestre Lab Research, Spain). The process of analyzing the metabolites used Cr as a standard reference.

### ASL

The parameter settings for 1 H-MRS were as follows: RT/TE = 4808/33 ms, FOV = 20 × 20 mm2, image size = 128 × 128, and slice = 1. The ASL perfusion image was obtained using Bruker Para Vision 360 software.

#### DTI

The DTI parameter settings include TR of 3,000 ms, TE of 19.6 ms, 85 gradient directions, diffusion gradient on time of 4 ms, FOV = 20 × 20 mm², 0.5 mm slice thickness, and a 256 × 256 matrix. DTI images were analyzed using ParaVision 360 V3.0 software (Bruker Biospin, Germany) and DSI Studio.

### HE staining

Samples from the ileum region were taken for histopathology, while the liver tissue was placed in neutral buffered formalin for fixation, with a slice thickness of 4 μm. Paraffin sections were then deparaffinized in water stained with Harris hematoxylin for 5–10 min for nuclear staining, followed by staining with eosin staining solution for 1–3 min for cytoplasmic staining using a dehydrated transparent mount. Intestinal villus length and crypt depth and liver tissue were measured from photomicrographs captured with an OLYMPUS eclipse inverted Microscope (OLYMPUS CKX43-LP) using image software.

#### Nissl staining

After routine treatment in paraffin, Sect. (8 μm) were obtained using a microtome (Leica Histoscore MULTICUT). Paraffin sections were then dewaxed to water. Subsequently, they were stained with methylene blue staining solution for 10 min, differentiated in Nicholas differentiation solution, and processed in ammonium molybdate solution for 1 min. Following this, sections were dehydrated and transparently sealed. Observation and photography were conducted using a panoramic scanner (Panoramic Scan).

#### BBB permeability

The four groups of mice were injected with 4% Evans blue (EB; 2 mL/kg, Aladdin) through their tail veins. After 3 h, the mouse brain was removed, and the tissue was cut and homogenized in an ice water bath at 1000 g. The homogenate was then centrifuged for 15 min, and 750 µL of the supernatant was collected. Acetone was added to the supernatant in a ratio of supernatant to acetone = 3:7, followed by incubation at room temperature for 24 h. Thereafter, the mixture was centrifuged at 2000 *g* for 15 min, and the supernatant was collected. The absorbance value of both the standard and the sample was read at 620 nm using a microplate reader, and the EB concentration was calculated accordingly.

#### ELISA

Blood and hippocampal tissue samples were collected from each mouse centrifuged at 3000 rpm for 10 min. Serum and hippocampal tissue samples were collected according to weight (mg) to volume (mL) ratio of 1:9, with the corresponding amount of normal saline added. Grinding beads were introduced, and the samples were thoroughly ground in a high-speed and low-temperature tissue grinder. Subsequently, centrifugation at 3000 rpm for 10 min was performed, and the supernatant was immediately tested following the instructions provided for the ammonia (A086-1-1, 202,305) and glutamine (F30107-A, 202,307) assays.

### Biochemical testing

After administering anesthesia, about 0.8 ml of blood from blood vessels is extracted from the heart, and the supernatant was obtained by centrifugation at 3000 rpm for 15 min at room temperature. Serum was used to measure liver function levels in mice. Alanine aminotransferase, aspartate aminotransferase, and total bilirubin levels were measured using an automatic biochemical analyzer (XN-530, Sysmex).Quality control testing is performed prior to the application of automatic biochemical analyze. After the application of automatic biochemical analyze quality control test is passed, the prepared samples are sampled for test, in this process, the machine takes about 25µL sample. After interrupting sampler mode, perform sample analysis.When analysis of the sample is completed, close the sampler cover and press the mode switch.

### Morris water maze (MWM)

The cognitive dysfunction test was conducted using the MWM [30, 31]. The apparatus had a diameter of 120 cm and a height of 45 cm, with the water temperature maintained at 20 ± 2 °C. The pool was divided into four quadrants of equal area, and the platform was placed at the center of the target quadrant. Each mouse in all groups was trained for four consecutive days (9–12 days after CLP) to find the platform and reach a plateau. The time taken by the mice to reach the platform was separately recorded after they entered the water in each quadrant, serving as the latency period. If the mouse failed to find the platform within 60 s, the experimenter guided it to the platform and allowed it to remain there for 10 s, with an escape latency of 60 s. In the probe test (13 days after CLP), mice were placed in water opposite the target quadrant, and the number of times they passed through the original plateau area within 60 s was recorded, along with the time spent in the target quadrant.

## (Novel object recognition) NOR

The NOR was used to evaluate the cognitive function of exploring new object abilities [32]. Before the experiment, the mouse was gently placed in a square box (50 × 50 × 40 cm^3^) for 3 min to acclimate to the environment. The box was equipped with a camera directly above it, connected to the Super Maze Animal Behavior Video Analysis System (Xinruan Information Technology Ltd, Shanghai, China).

Subsequently, the mice were randomly placed in the center of the arena, where two identical objects were explored for 5 min each (day 7 after CLP). On day 8 after CLP, the two familiar objects were replaced with a novel object, and the mice were allowed to explore for 5 min. The novel object and discrimination index [(time spent exploring the novel object/time spent exploring the two objects) × 100%] were recorded and analyzed.

### QT-PCR

Hippocampal samples were collected from the brain tissue, and cell experiments were performed using C8-D1A (mouse cerebellar astrocytes)/CL-0506. TRIzol lysate was added, and total RNA was extracted. RNA was synthesized using a ReverTra Ace qPCR RT Kit to synthesize cDNA. A denaturation buffer was added to the cell sample to induce cell lysis, followed by RNA extraction, precipitation, washing, solubilization, and assessment of RNA integrity. Real-time fluorescence quantification PCR was performed using specific primers. For the gene of interest, the upstream primers of GFAP were 5’- AACAACCTGGCTGCGTATAGAC − 3,’ and the downstream primers of GFAP: 5’- ATCTCCTCCTCCAGCGATTCAA − 3.’ Similarly, for AQP4, upstream primers were 5’- GCATCGCTAAGTCCGTCTTCT − 3,’ and the downstream primers were 5’- GAGGTGTGACCAGGTAGAGGAT − 3.’ For B-actin, the upstream primers were 5’- GTACTCTGTGTGGATCGGTGG − 3,’ and the downstream primers were 5’- GCAGCTCAGTAACAGTCCG − 3.’ The PCR protocol included pre-denaturation at 95 °C for 10 min, followed by denaturation at 95 °C for 15 s, annealing at 62 °C with elongation for 1 min, and a total of 40 cycles.

### Immunofluorescence

Briefly, the brain samples were fixed overnight in 4% paraformaldehyde and subjected to immunofluorescence staining. The astrocytes were pre-treated with NH_4_Cl (Chemuza, 12125-02-9) or TGN020 (Selleck, S0158). Primary astrocytes were then removed from the cell incubator and fixed in 4% paraformaldehyde after washing, followed by permeabilization in 0.3% TX-100 and blocking with 2% bovine serum albumin (BSA). The cells were treated with primary antibodies, including mouse anti-GFAP (1:300, Wuhan Mitaka, 60190-1-Ig), rabbit anti‐AQP4 (1:300, Wuhan Mitaka, 16473-1-AP) overnight at 4 °C. Subsequently, the cells were then incubated with secondary antibodies: goat anti-rabbit IgG/TRITC (1:100; Nakasugi Golden Bridge, ZF-0316) and goat anti-mouse IgG/FITC (1:100; Nakasugi Golden Bridge, ZF-0312). All images were acquired using a panoramic scanner (3DHISTECH Panoramic Scan). Positive fluorescence staining was calculated using image software.

#### Western blot analysis

Brain tissue and astrocyte samples were homogenized in RIPA buffer to extract the proteins. Different groups of brain tissues and astrocytes were treated with different drugs. The primary antibodies used included mouse anti-GFAP (1:10000, Wuhan Mitaka, 60190-1-Ig), rabbit anti‐AQP4 (1:15000, Wuhan Mitaka, 16473-1-AP), Mouse Anti-β-tubulin (1:2000, Nakasugi Kanabashi, TA-10). The sample‐loaded membranes were incubated overnight at 4 °C and then, post 8–12 h of incubation, treated with secondary antibody, Goat Anti-Rabbit IgG(1:5000, Nakasugi Golden Bridge, ZB-2301), and Goat Anti-MOUSE IgG(1:5000, Nakasugi Golden Bridge, ZB-2301). A gel imaging analysis system (Beijing Saizhi, Champchemi 610 plus) was used to detect band signals.

#### Astrocytes CCK8 assay

Astrocyte viability was determined using a CCK8 kit (BS350A, Biosharp). The cell suspension was seeded in a 96-well plate with 100 µL per well, containing 5,000 cells per well. Subsequently, 10 µL of CCK8 solution was added to each well, and the plate was further incubated in the cell culture incubator for 1–4 h.

## Astrocyte culture

The brain tissues of C57BL/6J mice were extracted and digested with 0.125% trypsin in Dulbecco’s modified Eagle’s medium (DMEM) in a cell culture incubator. DMEM containing 10% fetal bovine serum (FBS) was used to terminate the digestion. Single cells were seeded at a density of 1 × 10 cells/well in a 24-well culture plate pre-coated with polylysine, with a density of approximately 4 cm^-2^. After 72 h, the medium was replaced with a fresh, complete medium, and the cells were cultured for approximately 7 days to achieve 90% cell growth coverage. The plate was then spun at 260 rpm (24 h, 37 °C) to collect purified astrocytes [11].

### Statistical analysis

All data were expressed as the mean ± standard deviation (SD). GraphPad Prism 10.0.2 software (San Diego, CA, USA) was used for data analysis. Statistical significance was assessed using Student’s t-test, Mann–Whitney U test, ANOVA for multiple comparisons, two-way ANOVA with Tukey’s post hoc test, and one-way ANOVA with Tukey’s post hoc test. *p* < 0.05 was considered statistically significant.

### Electronic supplementary material

Below is the link to the electronic supplementary material.

Supplementary Material 1

Supplementary Material 2

## Data Availability

No datasets were generated or analysed during the current study.

## Abbreviations

SAE: Sepsis-associated encephalopathy
NHH: Non-hepatic hyperammonemia
CLP: Cecal ligation and perforation
ASL: Arterial spin labeling
DTI: Diffusion Tensor Imaging
1H-MRS: Proton magnetic resonance spectroscopy
AQP4: Aquaporin4channel
GFAP: Glial fibrillary acidic protein
KEGG: Kyoto encyclopedia of genes and genomes
GO: Gene ontology
PCoA: Principal coordinate analysis
LEfSe: LDA effect size
FA: Fractional anisotraphy
AD: Axial diffusion
RD: Radical diffusion
MD: Mean Diffusivity
Glu: Glutamate
Cr: Creatine
NAA: N-acetylaspartate
Glx: Glutamate + glutamine, T2WI: T2 weighted image
