# Supplementary material for: NHH promotes Sepsis-associated Encephalopathy with the expression of AQP4 in astrocytes through the gut-brain Axis
Source: J Neuroinflammation. 2024 May 27;21:138. doi: 10.1186/s12974-024-03135-2 (PMC11131257; doi:10.1186/s12974-024-03135-2)
Supplement: Supplementary file 1 — Supplementary Material 1 [file 12974_2024_3135_MOESM1_ESM.docx]

**Supplementary Materials 1** Comparison of the types of intestinal microbiota between the CLP group and the Sham group


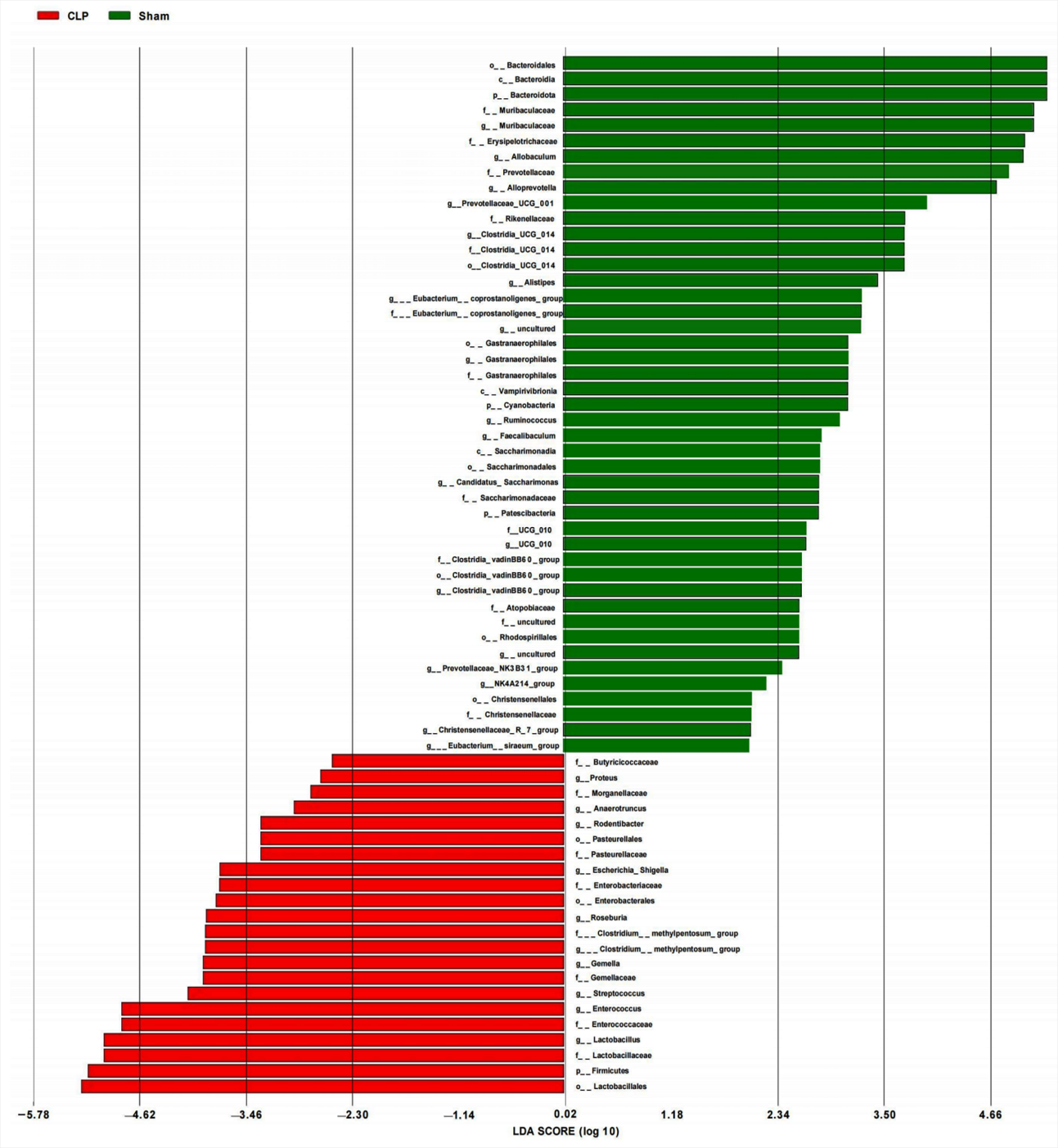


**Supplementary Materials 2** Comparison of the types of intestinal microbiota between the CLP group and the CLP+FMT group


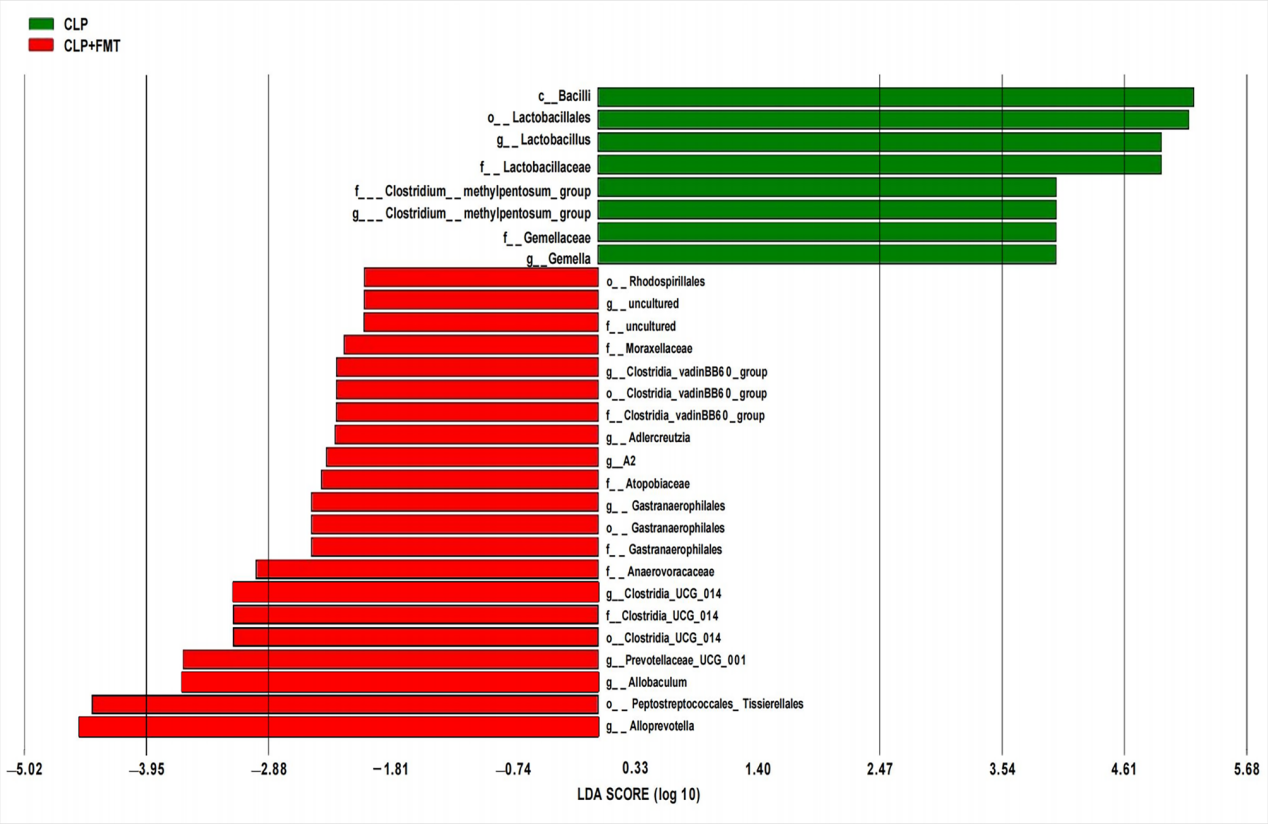


**Supplementary Materials 3** Rarefaction Curve of 16S rDNA


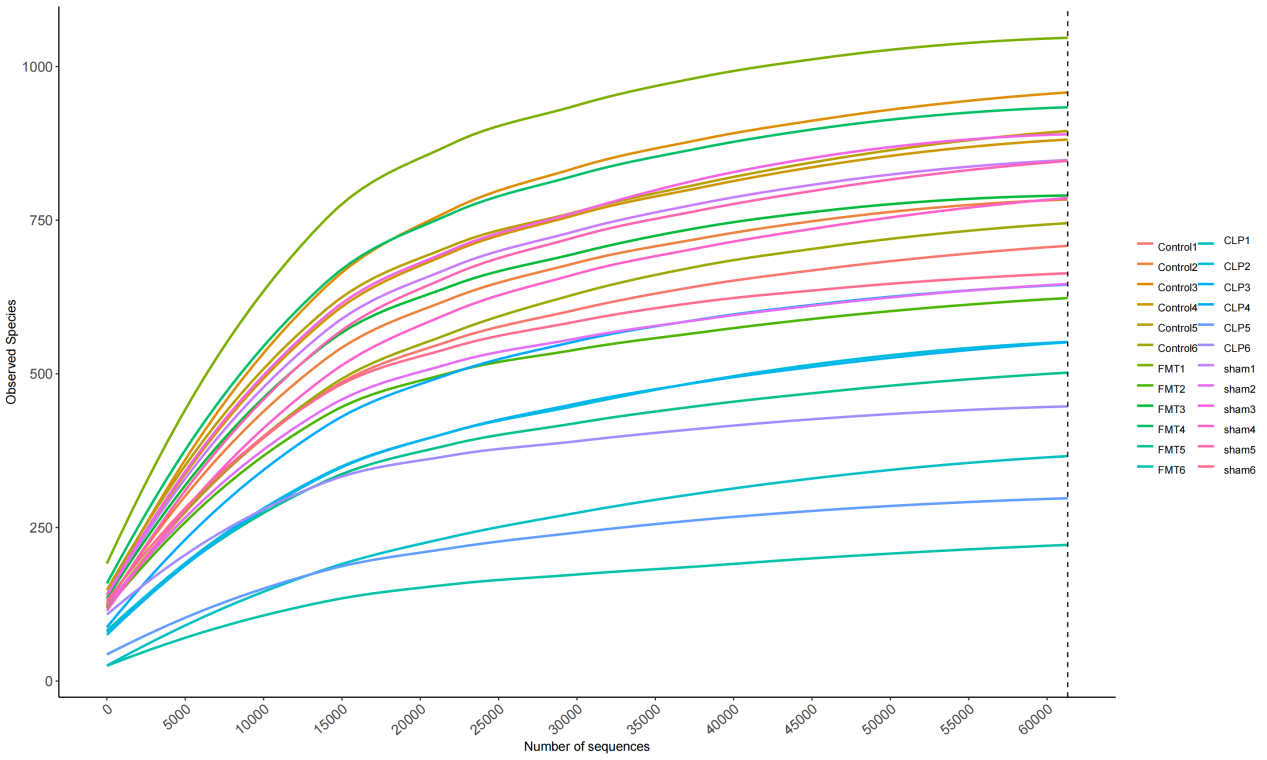


The rarefaction curve is a powerful tool that can effectively indicate the rationality of the sequencing data quantity and indirectly reflect the species abundance in the sample. A flat curve signifies that the sequencing data volume is appropriate, suggesting that additional data would yield only marginal increases in new Amplicon Sequence Variants (ASVs).

**Supplementary Materials 4**  Rank-abundance curves of 16S rDNA


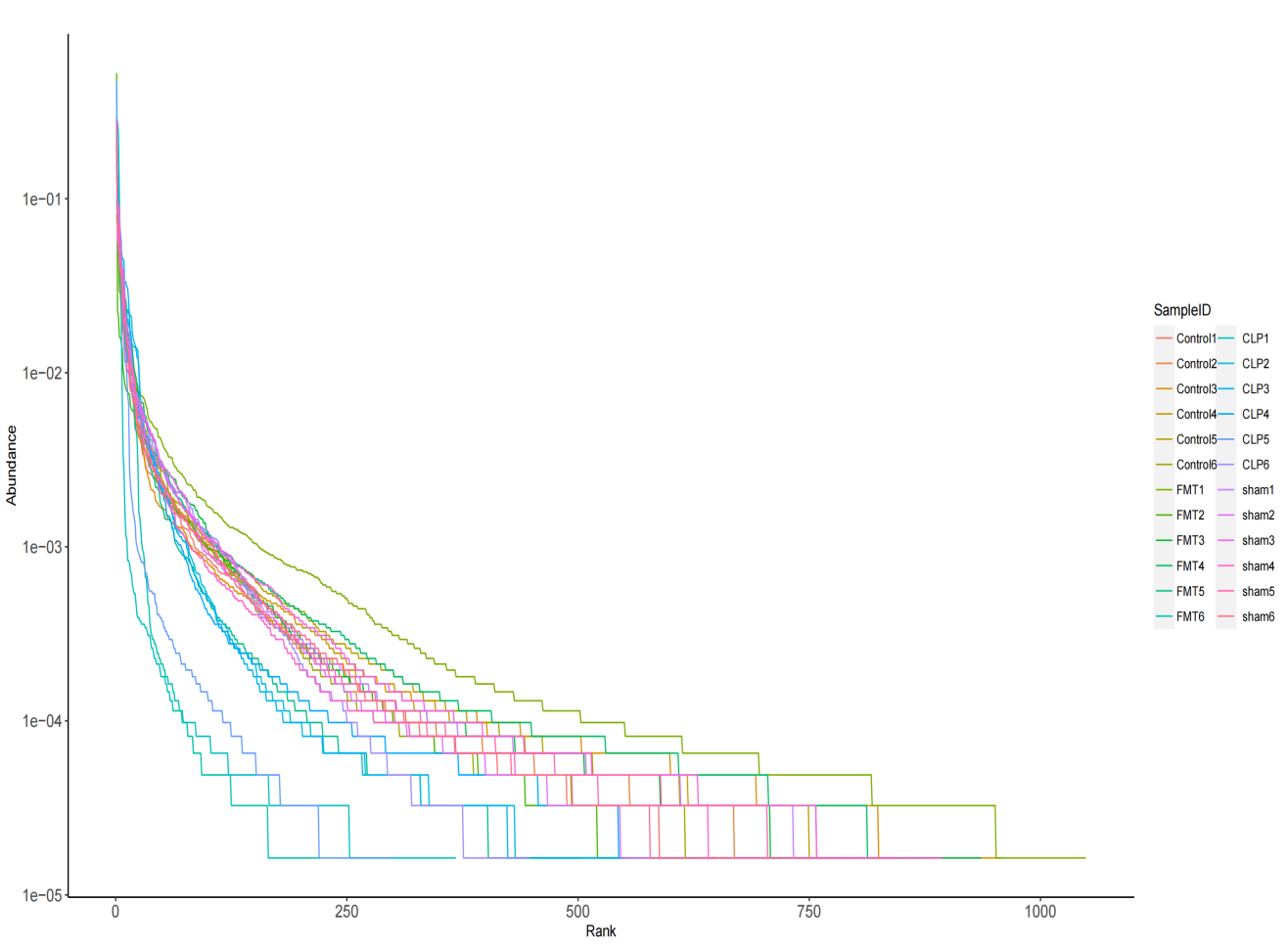


The width of the curve on the horizontal axis reflects the abundance of species in the sample, indicating that a greater abundance leads to a wider distribution on the horizontal axis. The smoothness of the curve is indicative of the homogeneity of species within the sample, with a flatter curve suggesting a more even distribution of species.

**Supplementary Materials 5** Shannon curves of 16S rDNA


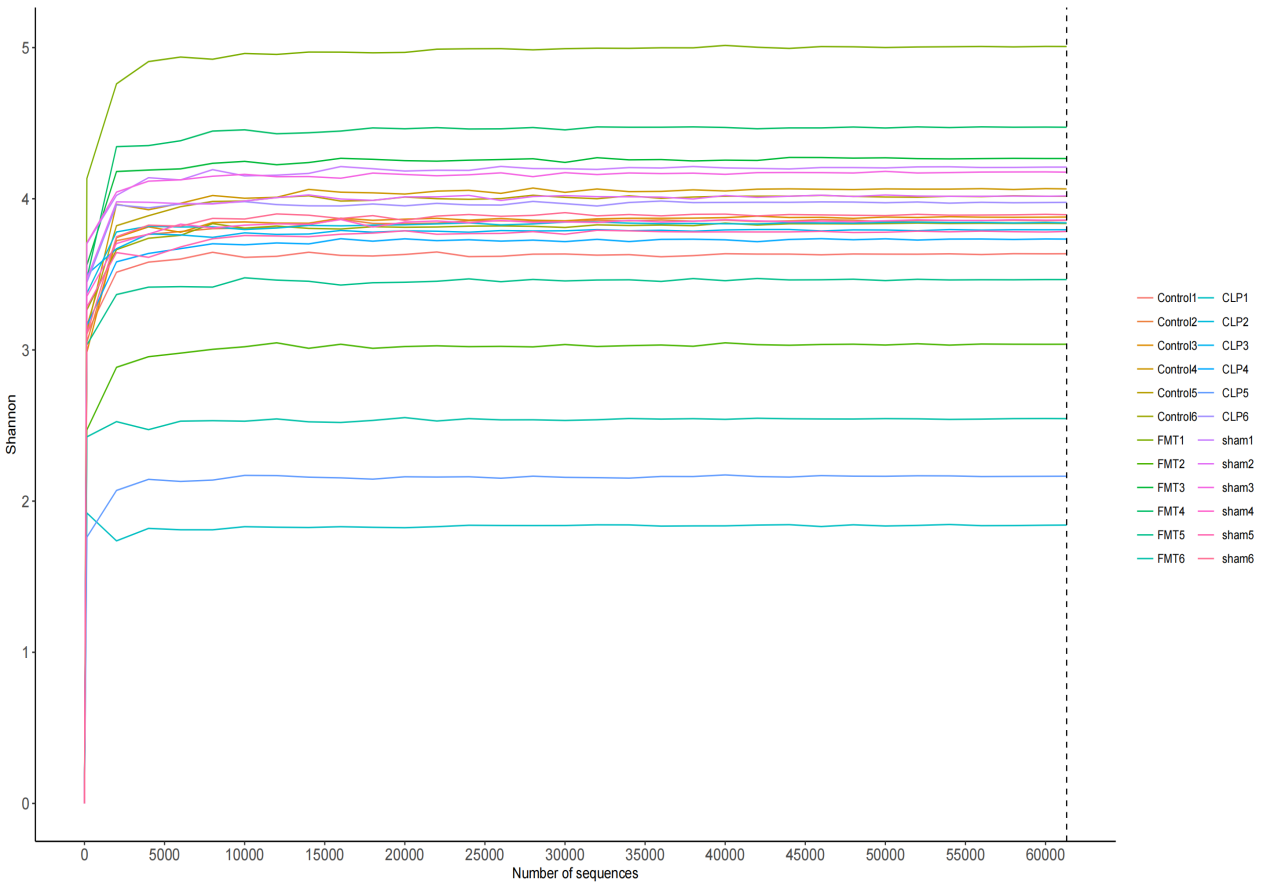


The Shannon curve is derived from the microbial diversity index of each sample at various sequencing depths. A flat curve suggests that the sequencing data is sufficiently large to capture most microbial information in the sample.

**Supplementary Materials 6** Species accumulation curves of 16S rDNA


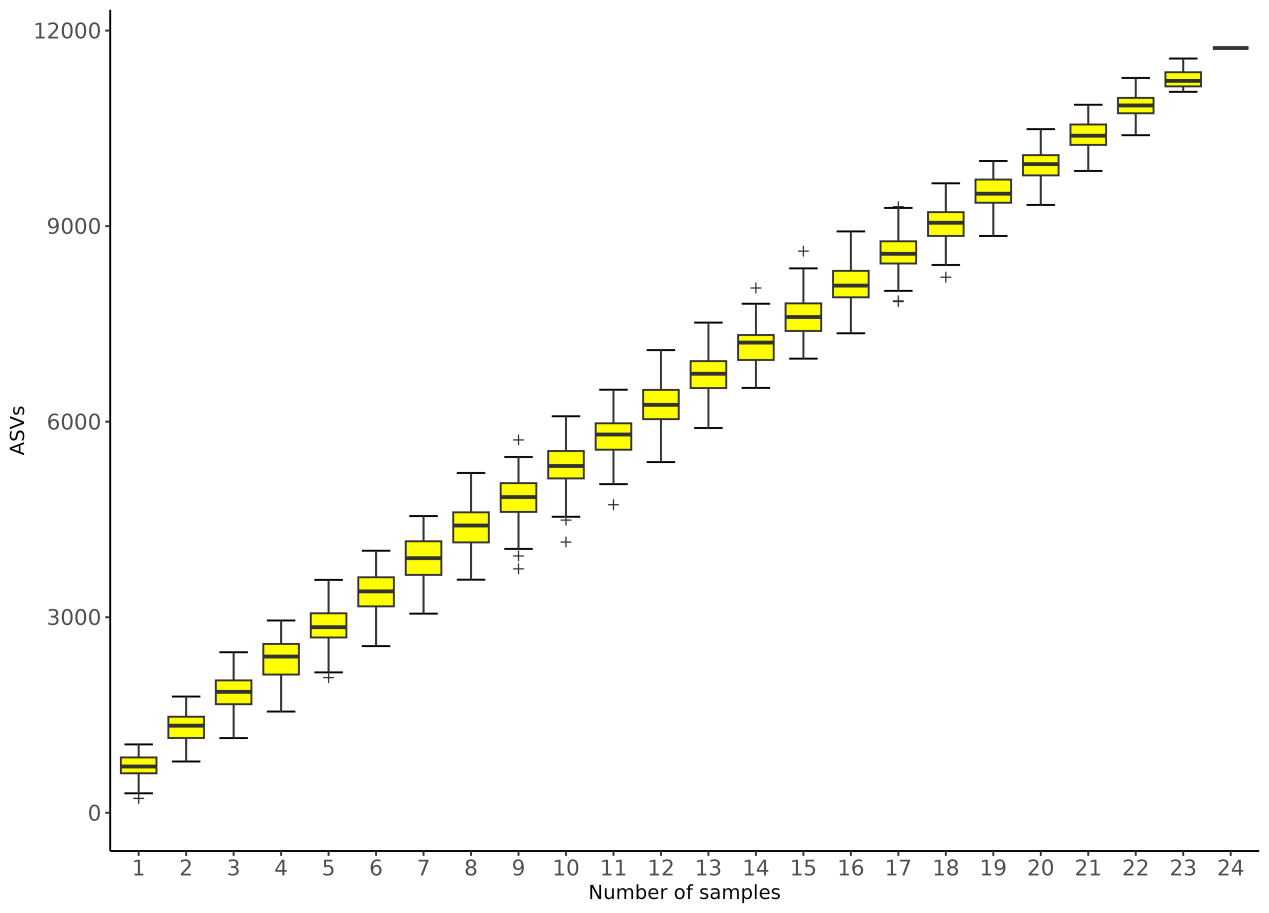


Species accumulation curves are effective tools for investigating the species composition of samples and predicting species abundance as the sample size increases. These analyses describe the increase in species diversity as the sample size increases, reflecting the rate of emergence of new ASVs (new species) under continuous sampling. An important observation is that within a certain range, if the position of the box plot increases sharply with the increase of the sample size, this indicates the discovery of a large number of species in the community.
